# Recovery of posterior communicating artery aneurysm induced oculomotor nerve palsy: a comparison between surgical clipping and endovascular embolization

**DOI:** 10.1186/s12883-020-01847-5

**Published:** 2020-09-18

**Authors:** Li-qiang Tian, Qing-xi Fu

**Affiliations:** 1 Department of Neurosurgery, Linyi People’s Hospital, Linyi, 276003 Shandong Province China; 2 Department of Neurology, Linyi People’s Hospital, Linyi, 276003 Shandong Province China

**Keywords:** Aneurysm, Nerve palsy, Surgical clipping, Endovascular embolization

## Abstract

**Background:**

Oculomotor nerve palsy (ONP) is a common symptom of posterior communicating artery aneurysm (PcomAA) that can lead to impaired eye movement and pupil dilation. Currently, surgical clipping and endovascular embolization are the two most popular treatment methods for PcomAA-induced ONP; however, the recovery outcome between the two methods remains to be elucidated.

**Methods:**

In the present study, we thoroughly compared the pretreatment factors and recovery outcome of the two treatments on 70 patients with PcomAA-induced ONP. The patients were separated into two groups based on the treatment that was received. Pretreatment factors, including age, sex, time period between ONP onset and treatment, ONP type, aneurysm diameter, status of subarachnoid hemorrhage and aneurysm rupture were recorded for each individual patient. Recovery outcome of the patients was assessed over a 12-month period.

**Results:**

No significant differences were observed in any of the analyzed factors. Importantly, we revealed a significantly higher full recovery rate for the patients receiving the surgical clipping treatment than the ones that received the endovascular embolization treatment. In addition, we showed that patients’ age was negatively correlated with the recovery extent in both treatment groups.

**Conclusions:**

The outcome of our study suggests that surgical clipping might be a better option to treat PcomAA-induced ONP.

## Highlights

- A total of 70 PcomAA-induced ONP patients were treated with either the surgical clipping or the endovascular embolization method.
- Baseline status of the patients was comparable between the two treatment groups.
- Patients receiving the surgical clipping treatment achieved a significantly higher full recovery rate compared to the endovascular embolization treatment.
- Age might be a prognostic factor for PcomAA-induced ONP full recovery

## Background

Posterior communicating artery (PcomA) is part of the circle of Willis that supplies blood to the brain and surrounding tissues, including floor of third ventricle, optic chiasm and tract, thalamus, hypothalamus, internal capsule and pituitary stalk. It connects the internal carotid artery to the anterior and middle cerebral arteries on the anterior side of the brain and communicates with the posterior cerebral artery on the posterior side. PcomA aneurysm (PcomAA) is the second most common intracranial aneurysm, accounting for approximately 25% of the total cases [1]. One of the most common consequence arisen from PcomAA is oculomotor nerve palsy (ONP) due to the increased compression from the enlarged PcomA [2]. A few mechanisms have been proposed for the PcomAA-induced ONP, including direct mass effect on the oculomotor nerve, pulsating effect from the aneurysm and subarachnoid hemorrhage (SAH)-induced nerve irritation [3, 4]. Ptosis, ophthalmoplegia and diplopia are common symptoms of ONP and treatment is required to prevent aneurysm rupture.

Surgical clipping is a standard treatment option for PcomAA-induced ONP, where a metal clip is placed at the weakened site of the blood vessel via a small incision on the skull [1]. Endovascular embolization has become a popular alternative since its successful application on the International Subarachnoid Aneurysm Trial [5]. A spring-shaped coil made of platinum material is delivered to the aneurysm by a catheter via a groin artery under the guidance of X-ray. Although extensive studies have been carried out to determine which one of the two methods is the better option for ONP treatment [4, 6–11], controversy still exists in the field.

In the present study, we performed a retrospective analysis on surgical clipping or endovascular embolization treated PcomAA patients with ONP to assess the efficacy of the two methods on the functional recovery of PcomA-induced ONP.

## Methods

### Study design

The study protocol was approved by the local institutional review board of the Linyi People’s Hospital. Verbal consent was obtained from all patients for participating the study at the time of diagnosis.

The study was carried out on patients treated between 2015 and 2018 at Linyi People’s Hospital. A total of 70 patients with PcomAA-induced ONP were included in the study. Aneurysm treatment followed the standard recommendation from the International Study of Unruptured Intracranial Aneurysms [12]. Each patient was individually evaluated by the neurointerventional physicians and neurosurgeons for risk assessment based on their characteristics. The entire procedure of the two treatment methods was carefully explained to the patients by the doctor. If the patient did not have a preference, they will be randomly assigned to the two groups. Otherwise, their preferred treatment method was used. Patients with SAH symptom were intravenously administered with nimodipine and tranexamic acid on a daily basis before the treatment after admission.

The inclusion criteria were patients with PcomAA-induced ONP, treated with either the surgical clipping or the endovascular embolization methods. In addition, only patients who had at least one postoperative angiographic image taken and were followed for 12 months were included in this study. On the other hand, patients with diabetic ophthalmoplegia and/or hypertension and patients followed for less than 12 months were excluded from the study.

Basic patient characteristics, such as age, gender and time to treatment were recorded for each individual patient (Tables S1). In addition, ONP and aneurysm-related characteristics, including ONP type, aneurysm diameter, SAH grade, presence of aneurysm rupture were also individually recorded (Table S1). All characteristics were selected based on previously published similar studies [13–16].

### Treatment procedures

Surgical clipping was performed according to a previously described pterional approach [17]. Endovascular embolization was performed with coil packing according to previously reported guidance [18].

### Patient follow-up

The patients were monitored on a daily basis after the treatment and further followed for a 12-month period after being discharged from the hospital to assess their recovery state. At the end of the 12-month follow-up period, the patient was defined to be fully recovered if all the diagnostic symptoms of ONP was disappeared. If 1 or more symptoms remained, a partial recovery was defined for the patient. We followed the previously published diagnostic criteria for ONP [19], including ipsilateral ptosis, ophthalmoplegia, diplopia, mydriasis and impaired pupillary light reflex. The time to full recovery was recorded on the day of assessment when all symptoms were eliminated. Degree of ONP was evaluated by professional neuro-ophthalmologists, who were blinded for the treatment modality.

### Statistical analysis

SPSS software was used for statistical analysis. Unpaired t test and rank sum test were respectively used for parametrically and non-parametically distributed data to compare the variables between the two groups, where *p* < 0.05 was considered as statistical significance. Multivariate logistic regression analysis was used to examine the pretreatment factors that predict the surgical clipping and endovascular embolization outcome.

## Results

### Patient characteristics before treatment

The 70 patients with PcomAA-induced ONP were selected from a total of 713 intracranial aneurysm cases treated at our hospital. PcomAA was confirmed by the dual usage of computed tomography angiography and digital subtraction angiography. The patients were separated into two groups based on the treatment they received: the surgical clipping group consisted of 31 patients and the endovascular embolization group consisted of 39 patients.

To compare the characteristics of patients between the clipping and embolization groups, we first performed univariate analysis on gender, aneurysm diameter, time to treatment, ONP type, percentage of patients with SAH and percentage of patients with aneurysm rupture. No significant differences were observed in any of these characteristics (Table 1). We further performed multivariate logistic regression analysis on these characteristics and observed the same prediction of outcome (Table 2). Taken together, these results suggest that all the typical patient characteristics between the two treatment groups are comparable prior to the treatment.

**Table 1:** Comparison of the pretreatment factors between the two groups

Characteristic	SC group (*n* = 31)	EE group (*n* = 39)	*P* value
Sex (male:female)	14:17	21:18	*P* = 0.4356
Aneurysm diameter (mm)	7.6 ± 2,9	7.9 ± 3,0	*P* = 0.6891
time period between ONP onset and treatment (day)	28,2 ± 13.0	23.2 ± 14.7	*P* = 0.1386
ONP type (CO:PO)	23:8	32:7	*P* = 0.1542
% of SAH	6.5% (2/31)	12.8% (5/39)	
% of aneurysm rupture	9.7% (3/31)	10.1% (4/39)	

*CO* complete ONP, *PO* partial ONP, *SC* surgical clipping, *EE* endovascular embolization

**Table 2:** Multivariate logistic regression analysis of the pretreatment factors between the two groups

Characteristic	Regression coefficient	Standard error	*P*-value	OR (95% CI)
Age	−0.036	1.232	0.854	0.758 (0.324–8.546)
Sex (male:female)	−0.215	0.912	0.456	0.832 (0.467–6.435)
Aneurysm diameter (mm)	−0.463	0.253	0.786	0.764 (0.896–6.854)
time period between ONP onset and treatment (day)	−0.089	0.575	0.689	0.908 (1.121–7.549)
ONP type (CO:PO)	−2.432	0.934	0.965	0.674 (0.954–6.794)
% of SAH	−0.768	0.345	0.435	0.865 (1.134–7.658)
% of aneurysm rupture	−0.203	0.635	0.768	0.793 (1.675–8.954)

*CO* complete ONP, *PO* partial ONP

Among all the symptoms of ONP, ipsilateral ptosis was the most common one that were observed in 86% (60 out of 70) of the patients. There were 2 and 5 cases of SAH in the surgical clipping and the endovascular embolization groups, respectively (Table S1). Aneurysm rupture was observed in 3 and 4 patients of the two groups, respectively (Table S1).

### Patient recovery

A significant difference in the rate of full recovery was observed between the two groups. A 84.0% full recovery rate (26 out of 31) was achieved by patients in the surgical clipping group, whereas patients in the endovascular embolization group only reached a full recovery rate of 64.1% (25 out of 39) (Table 3). Statistical significance (*P* = 0.0154) was detected for the full recovery rate between the two groups (Table 3). However, no difference was observed for the average time period to full recovery between the two groups, likely due to the relatively large standard deviation values (Table 3).

**Table 3:** Comparison of the recovery outcomes between the two groups

	SC group (*n* = 31)	EE group (*n* = 39)	*P* value
Recovery status (FR:PR)	26:5	25:14	P = 0.0154
time period to full recovery (day)	118.4 ± 51.8	107.0 ± 54.1	*P* = 0.3757

*FR* full recovery, *PR* partial recovery, *SC* surgical clipping, *EE* endovascular embolization

**Fig. 1:**
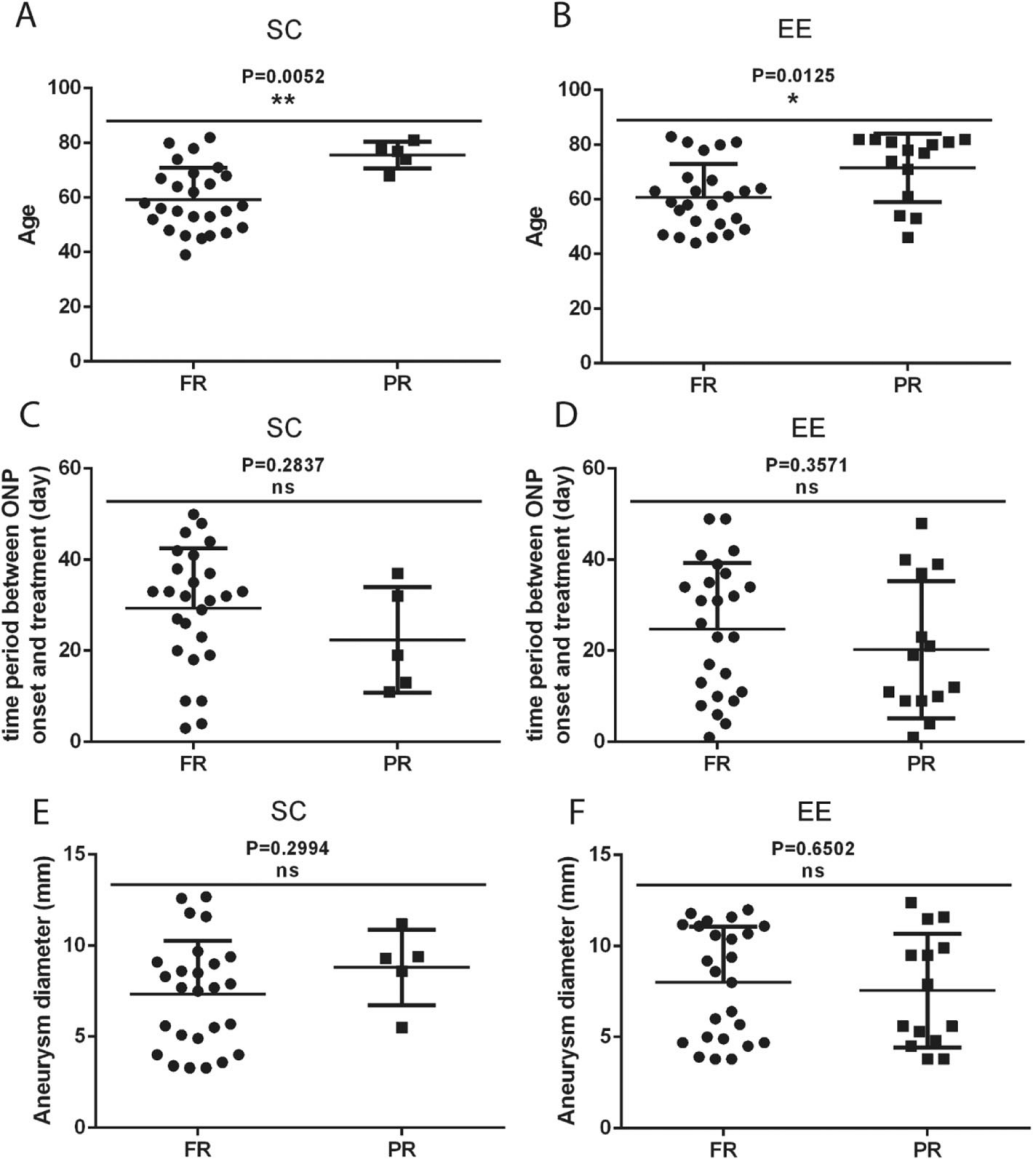
Comparison of age (**a**-**b**), length of pretreatment period (**c**-**d**) and aneurysm diameter (**e**-**f**) between the surgical clipping (SC) and endovascular embolization (EE) treatment groups. FR, full recovery; PR, partial recovery

To seek the potential causes that influence the full recovery rate, we analyzed the distribution of various prognostic factors in the fully and partially recovered patients of the two treatment groups. We found that there was a significant age difference between patients that were fully or partially recovered in both treatment groups, where the partially recovered patients were significantly older than the fully recovered ones (Fig. 1a-b). No differences were observed for other factors, such as length of the pretreatment period and aneurysm diameter (Fig. 1c-f).

In addition, we also checked the remaining ONP symptoms in patients who achieved partial recovery. We found that ipsilateral ptosis was the symptom that had the highest rate of recovery, while the impaired pupillary light reflex symptom had the lowest rate of recovery (Table 4). Other symptoms, such as ophthalmoplegia, diplopia and mydriasis had similar recovery rate (Table 4). In general, no difference was observed in any of the remaining symptoms between the clipping and embolization groups.

**Table 4:** Comparison of the remaining incompletely recovered ONP symptoms between the two groups

Incomplete ONP symptoms	SC group (*n* = 5)	EE group (*n* = 14)
ipsilateral ptosis	1 (20%)	2 (14.3%)
ophthalmoplegia	3 (60%)	8 (57.1%)
diplopia	2 (40%)	9 (64.3%)
mydriasis	4 (80%)	9 (64.3%)
impaired pupillary light reflex	5 (100%)	11 (78.6%)

*SC* surgical clipping, *EE* endovascular embolization

## Discussion

Surgical clipping and endovascular embolization are the two most common treatment options for aneurysm-induced ONP. However, a long debate has been going on in the clinical field to determine which method has more advantages. Here, we performed a retrospective study to examine the recovery status of 70 PcomAA-induced ONP patients treated by the two methods. We showed that patients receiving the surgical clipping treatment achieved a significantly higher rate of full recovery than the patients receiving the endovascular embolization treatment, despite of a comparable time period to full recovery. This favors surgical clipping as a preferred method to treat PcomAA-induced ONP. In addition, we found that age of the patient was negatively correlated with the recovery extent. Our observation agrees with some of the previous studies that analyzed the treatment of ONP secondary to PcomAA with the clipping and coiling methods [13, 16], although a recently published study revealed no significant difference between the two [14].

In the present study, patients’ background characteristics and prognostic factors were comparable between the SC and EE treatment groups. Therefore, a relatively fair comparison was carried out between the two groups. Previous studies have reported that the full recovery rates of ONP after the surgical clipping and endovascular embolization treatments fall between 30 and 86%, and 0 and 100%, respectively [2, 3, 7, 20, 21]. We achieved 84.0 and 64.1% full recovery rate for the two treatments, both of which are within the reported ranges and well above many of the previous reports. Consistent with previous studies [15], all patients diagnosed with partial ONP, except 1 from each treatment group, achieved full recovery. The two partially recovered patients diagnosed with partial ONP were both above the age of 80, which might account for their decreased recovery extent. This is supported by our observation that partially recovered patients were significantly older than the fully recovered ones in both treatment groups.

There are a few limitations with this study. First, we cannot perform a randomized study since we have to follow the patients’ final decision on the choice of treatment. In addition, the number of patient with PcomAA-induced ONP is rather limited. Bigger sample size would provide more reliable insights on the outcomes of the two treatments. Moreover, all patients were treated in a single hospital (Linyi People’s Hospital), which might generate some potential biases to the analysis. Inclusion of patients from multiple hospitals would cover different treatment environments to minimize the chances of such biases.

## Conclusion

In summary, our results indicate that surgical clipping might be a better treatment for PcomAA-induced ONP in term of the recovery extent.

## Supplementary information

**Additional file 1: Table S1.** Characteristics of the 70 participated patients. CO, complete ONP; PO, partial ONP; FR, full recovery; PR, partial recovery; SC, surgical clipping; EE, endovascular embolization.

## Data Availability

All data generated or analysed during this study are included in this published article and its supplementary information files.

## Abbreviations

ONP: Oculomotor nerve palsy
PcomAA: Posterior communicating artery aneurysm
PcomA: Posterior communicating artery
