# Supplementary material for: Recovery of posterior communicating artery aneurysm induced oculomotor nerve palsy: a comparison between surgical clipping and endovascular embolization
Source: BMC Neurol. 2020 Sep 18;20:351. doi: 10.1186/s12883-020-01847-5 (PMC7501645; doi:10.1186/s12883-020-01847-5)
Supplement: Supplementary file 1 — Additional file 1: Table S1. Characteristics of the 70 participated patients. CO, complete ONP; PO, partial ONP; FR, full recovery; PR, partial recovery; SC, surgical clipping; EE, endovascular embolization. [file 12883_2020_1847_MOESM1_ESM.docx]

**Table S1: Characteristics of the 70 participated patients.**

CO, complete ONP; PO, partial ONP; FR, full recovery; PR, partial recovery; SC, surgical clipping; EE, endovascular embolization.

| Patient No. | Age | Sex | time period between ONP onset and treatment (day) | ONP type | Aneurysm diameter (mm) | SAH grade | Aneurysm rupture | Recovery status | time period to full recovery (day) | Treatment group |
| --- | --- | --- | --- | --- | --- | --- | --- | --- | --- | --- |
| 1 | 40-50 | M | 20 | CO | 4,0 | 0 | no | FR | 103 | SC |
| 2 | 50-60 | M | 31 | CO | 4,0 | 0 | no | FR | 60 | SC |
| 3 | 50-60 | M | 9 | CO | 9,7 | 0 | no | FR | 196 | SC |
| 4 | 60-70 | M | 37 | CO | 5,5 | 0 | no | PR | NA | SC |
| 5 | 60-70 | M | 46 | PO | 8,5 | 0 | no | FR | 177 | SC |
| 6 | 70-80 | M | 32 | CO | 9,4 | 1 | yes | PR | NA | SC |
| 7 | 70-80 | M | 35 | PO | 5,7 | 0 | no | FR | 55 | SC |
| 8 | 80-90 | M | 13 | PO | 9,3 | 0 | no | PR | NA | SC |
| 9 | 40-50 | M | 44 | CO | 7,7 | 0 | no | FR | 77 | SC |
| 10 | 60-70 | M | 37 | CO | 9,4 | 0 | no | FR | 156 | SC |
| 11 | 50-60 | M | 23 | CO | 5,1 | 0 | no | FR | 197 | SC |
| 12 | 70-80 | M | 11 | CO | 11,2 | 2 | yes | PR | NA | SC |
| 13 | 80-90 | M | 42 | PO | 3,4 | 0 | no | FR | 152 | SC |
| 14 | 40-50 | M | 38 | CO | 12,6 | 0 | no | FR | 90 | SC |
| 15 | 80-90 | F | 48 | PO | 7,7 | 0 | no | FR | 190 | SC |
| 16 | 70-80 | F | 4 | CO | 3,3 | 0 | no | FR | 196 | SC |
| 17 | 70-80 | F | 50 | CO | 12,7 | 0 | no | FR | 28 | SC |
| 18 | 60-70 | F | 27 | CO | 9,0 | 0 | no | FR | 93 | SC |
| 19 | 60-70 | F | 19 | CO | 5,6 | 0 | no | FR | 143 | SC |
| 20 | 60-70 | F | 9 | CO | 11,6 | 0 | no | FR | 188 | SC |
| 21 | 50-60 | F | 32 | PO | 3,3 | 0 | no | FR | 91 | SC |
| 22 | 50-60 | F | 26 | PO | 11,8 | 0 | no | FR | 150 | SC |
| 23 | 50-60 | F | 29 | CO | 7,5 | 0 | yes | FR | 100 | SC |
| 24 | 70-80 | F | 19 | CO | 8,6 | 0 | no | PR | NA | SC |
| 25 | 50-60 | F | 33 | CO | 9,1 | 0 | no | FR | 133 | SC |
| 26 | 60-70 | F | 32 | CO | 7,9 | 0 | no | FR | 53 | SC |
| 27 | 30-40 | F | 33 | CO | 8,6 | 0 | no | FR | 46 | SC |
| 28 | 40-50 | F | 41 | CO | 4,9 | 0 | no | FR | 72 | SC |
| 29 | 40-50 | F | 3 | CO | 5,5 | 0 | no | FR | 134 | SC |
| 30 | 40-50 | F | 18 | CO | 3,6 | 0 | no | FR | 86 | SC |
| 31 | 50-60 | F | 33 | PO | 8,3 | 0 | no | FR | 54 | SC |
| 32 | 60-70 | M | 8 | PO | 11,1 | 0 | no | FR | 134 | EE |
| 33 | 60-70 | M | 1 | CO | 11,8 | 0 | no | FR | 151 | EE |
| 34 | 50-60 | M | 34 | CO | 11,4 | 0 | no | FR | 147 | EE |
| 35 | 80-90 | M | 23 | CO | 5,3 | 2 | yes | PR | NA | EE |
| 36 | 50-60 | M | 37 | CO | 10,4 | 0 | no | FR | 36 | EE |
| 37 | 60-70 | M | 35 | CO | 11,6 | 0 | no | FR | 159 | EE |
| 38 | 60-70 | M | 31 | CO | 11,1 | 0 | no | FR | 123 | EE |
| 39 | 60-70 | M | 23 | CO | 3,8 | 0 | no | FR | 103 | EE |
| 40 | 40-50 | M | 4 | PO | 4,7 | 0 | no | FR | 133 | EE |
| 41 | 60-70 | M | 10 | CO | 9,5 | 0 | no | PR | NA | EE |
| 42 | 80-90 | M | 11 | PO | 9,9 | 0 | no | PR | NA | EE |
| 43 | 80-90 | M | 6 | CO | 3,9 | 0 | no | FR | 103 | EE |
| 44 | 50-60 | M | 39 | CO | 6,0 | 0 | no | FR | 69 | EE |
| 45 | 50-60 | M | 49 | CO | 12,0 | 0 | no | FR | 141 | EE |
| 46 | 80-90 | M | 11 | CO | 9,2 | 0 | no | FR | 94 | EE |
| 47 | 70-80 | M | 39 | CO | 4,5 | 2 | yes | PR | NA | EE |
| 48 | 40-50 | M | 48 | CO | 3,8 | 0 | no | PR | NA | EE |
| 49 | 80-90 | M | 21 | CO | 3,8 | 0 | no | PR | NA | EE |
| 50 | 40-50 | M | 26 | PO | 9,4 | 0 | no | FR | 50 | EE |
| 51 | 40-50 | M | 42 | PO | 4,7 | 0 | no | FR | 43 | EE |
| 52 | 70-80 | M | 15 | PO | 5,6 | 0 | no | PR | NA | EE |
| 53 | 70-80 | F | 4 | CO | 5,6 | 0 | no | PR | NA | EE |
| 54 | 50-60 | F | 37 | CO | 11,5 | 0 | no | PR | NA | EE |
| 55 | 70-80 | F | 40 | CO | 8,6 | 0 | no | FR | 35 | EE |
| 56 | 40-50 | F | 34 | CO | 11,2 | 0 | no | FR | 23 | EE |
| 57 | 40-50 | F | 10 | CO | 4,9 | 0 | no | FR | 179 | EE |
| 58 | 80-90 | F | 9 | CO | 11,6 | 1 | yes | PR | NA | EE |
| 59 | 80-90 | F | 23 | CO | 8,0 | 0 | no | FR | 43 | EE |
| 60 | 60-70 | F | 49 | CO | 3,8 | 0 | no | FR | 32 | EE |
| 61 | 80-90 | F | 32 | PO | 5,7 | 0 | no | FR | 26 | EE |
| 62 | 50-60 | F | 9 | CO | 4,8 | 0 | no | PR | NA | EE |
| 63 | 80-90 | F | 1 | CO | 7,9 | 1 | yes | PR | NA | EE |
| 64 | 50-60 | F | 17 | CO | 5,0 | 0 | no | FR | 39 | EE |
| 65 | 50-60 | F | 31 | CO | 10,7 | 0 | no | FR | 88 | EE |
| 66 | 80-90 | F | 12 | CO | 9,5 | 0 | no | PR | NA | EE |
| 67 | 50-60 | F | 13 | CO | 6,4 | 0 | no | FR | 42 | EE |
| 68 | 40-50 | F | 9 | CO | 10,6 | 0 | no | FR | 149 | EE |
| 69 | 60-70 | F | 41 | CO | 4,5 | 0 | no | FR | 187 | EE |
| 70 | 70-80 | F | 19 | CO | 12,4 | 1 | no | PR | NA | EE |
